# Survival Outcomes in Metaplastic Versus Triple-Negative Invasive Breast Carcinoma: A Propensity Score-Matched Study

**DOI:** 10.3390/medicina62101944

**Published:** 2026-10-08

**Authors:** Burcu Caner, Hulya Ertas, Sibel Oyucu Orhan, Seher Bahar

**Affiliations:** 1 Medical Oncology, Aydin Ataturk State Hospital, Aydin 09020, Türkiye; 2 Medical Oncology, Ali Osman Sonmez Oncology Hospital, Bursa 16040, Türkiye; 3 Medical Oncology, Bursa City Hospital, Bursa 16110, Türkiye; 4 Radiation Oncology, Balikesir Ataturk City Hospital, Balikesir 10100, Türkiye

**Keywords:** metaplastic breast carcinoma, triple-negative breast cancer, propensity score matching, overall survival, neutrophil-to-lymphocyte ratio

## Abstract

*Background and Objectives*: Metaplastic breast carcinoma (MBC) is a rare, predominantly triple-negative breast cancer subtype with aggressive clinical behaviour. This study compared the clinicopathological characteristics and survival outcomes of MBC with triple-negative invasive breast carcinoma (TN-IBC) and assessed the prognostic value of the neutrophil-to-lymphocyte ratio (NLR). *Materials and Methods*: Patients with stage I–III MBC or TN-IBC diagnosed between January 2003 and January 2024 were retrospectively reviewed. Among 35 patients with MBC and 103 with TN-IBC, 1:1 propensity score matching by age and TNM stage generated two groups of 35 patients. Disease-free survival (DFS) and overall survival (OS) were evaluated using Kaplan–Meier estimates and Cox proportional hazards models with robust sandwich variance clustered by matched pair. *Results*: All 70 matched patients had triple-negative disease. During a median follow-up of 7.7 years, recurrence occurred in 22.9% of patients with MBC and 17.1% of those with TN-IBC, while mortality rates were 25.7% and 8.6%, respectively. Five-year DFS rates were 73.9% and 85.2%, respectively; however, MBC was not significantly associated with DFS (HR = 1.70, 95% CI 0.67–4.30; *p* = 0.262). Five-year OS rates were 79.8% and 94.3%, respectively. Metaplastic histology was associated with a higher mortality hazard (HR = 3.53, 95% CI 1.01–12.41; *p* = 0.049). Median NLR was higher in MBC than in TN-IBC (2.31 vs. 1.85; *p* = 0.019). High NLR was associated with poorer OS in the overall matched cohort (HR = 3.36, 95% CI 1.07–10.57; *p* = 0.038), although NLR was not significantly associated with survival when analysed as a continuous variable or within the MBC subgroup. *Conclusions*: MBC was associated with inferior OS compared with TN-IBC after propensity score matching and pair-adjusted survival analysis, while DFS did not differ significantly. Elevated NLR was associated with poorer OS in the overall matched cohort but was not validated as an MBC-specific prognostic biomarker.

## 1. Introduction

Breast cancer is a heterogeneous disease with diverse histopathologic subtypes and molecular profiles that significantly influence clinical behavior, treatment response, and patient outcomes. Invasive breast carcinoma of no special type (IBC-NST) represents the most common form and is typically classified according to estrogen receptor (ER), progesterone receptor (PR), and human epidermal growth factor receptor 2 (HER2) status. These molecular subtypes, including luminal A, luminal B, HER2-enriched, and triple-negative breast cancer (TNBC), provide a framework for prognostication and therapeutic strategies, with TNBC accounting for approximately 10–20% of IBC-NST cases and generally associated with a poorer prognosis [1,2,3,4].

Metaplastic breast carcinoma (MBC) is an uncommon and biologically aggressive variant of invasive breast carcinoma, accounting for less than 1% of all breast cancers. According to the 2019 World Health Organization (WHO) classification, MBC encompasses a spectrum of tumors characterized by non-glandular differentiation into squamous or mesenchymal components, including spindle, chondroid, or osseous elements. Subtypes include squamous cell carcinoma, spindle cell carcinoma, metaplastic carcinoma with mesenchymal differentiation, mixed metaplastic carcinoma, fibromatosis-like carcinoma, and low-grade adenosquamous carcinoma. Among these, squamous and spindle cell carcinoma are the most frequent, while fibromatosis-like and low-grade adenosquamous variants are exceedingly rare [5,6,7].

Clinically, MBC often presents as a rapidly enlarging, high-grade tumor with a tendency for hematogenous spread, particularly to the lungs, and relatively low rates of axillary lymph node involvement. Despite sharing the triple-negative receptor profile with TN-IBC, MBC is distinct in its morphology, metastatic pattern, and clinical course. Several studies have demonstrated that MBC is associated with worse disease-free and overall survival compared to TN-IBC, even after controlling for stage and receptor status. Importantly, MBC also demonstrates substantial chemoresistance, with reported response rates to standard neoadjuvant chemotherapy as low as 10–15%, markedly inferior to the 30–40% pathologic complete response rates observed in TN-IBC [8,9].

The Nottingham Prognostic Index (NPI) is a clinicopathological composite score that integrates tumour size, lymph-node involvement, and histological grade. Developed and validated in operable breast cancer, it stratifies long-term survival more effectively than any of its individual components, with higher scores indicating a less favourable prognosis [10]. The NPI has also retained prognostic value in triple-negative breast cancer, although the biological heterogeneity of triple-negative tumours and rare histological variants may limit the extent to which a conventional anatomical and pathological index captures subtype-specific risk [11]. Subsequent evidence demonstrates that NPI independently predicts both OS and DFS in the triple-negative breast cancer subgroup [12]. Because MBC commonly has a triple-negative phenotype but is biologically distinct from TN-IBC, comparison of NPI values between these groups may help determine whether conventional clinicopathological risk burden accounts for differences in outcome.

Systemic inflammation has increasingly been recognised as an important contributor to breast cancer progression and prognosis. The neutrophil-to-lymphocyte ratio (NLR), which can be readily calculated from a routine complete blood count, is an inexpensive and reproducible marker reflecting the balance between systemic inflammation and host immune activity. Previous studies have associated an elevated pretreatment NLR with poorer disease-free and overall survival in breast cancer, with particularly pronounced associations reported in triple-negative breast cancer [13,14,15]. Nevertheless, the prognostic utility of NLR remains controversial, as a recent large study of early breast cancer found no independent association between NLR and survival or consistent correlation with tumour immune characteristics [16]. Given that MBC frequently exhibits a triple-negative phenotype but has distinct biological and clinical characteristics, evidence regarding the prognostic significance of NLR specifically in MBC remains limited. Therefore, evaluating NLR may help clarify whether systemic inflammatory status contributes to the survival differences between MBC and TN-IBC.

Given its rarity, histologic heterogeneity, and poor treatment response, MBC represents a major clinical challenge. Accurate differentiation from TN-IBC is essential for prognostication and therapeutic decision-making. In our study, we aimed to compare clinicopathological features and survival outcomes of MBC with TN-IBC using a propensity score-matched (PSM) design.

## 2. Materials and Methods

This study included breast cancer patients older than 18 years. We retrospectively analyzed patient records of patients diagnosed with early-stage breast cancer (stages 1, 2, and 3) from 1 January 2003, to 1 January 2024. Demographic characteristics, pathological (ER, PR, HER2, grade, Ki67), and clinical data (type of surgery, radiotherapy, chemotherapy, recurrence, metastasis, and date of death) were recorded. Patients with metaplastic histology and triple-negative invasive breast carcinoma histology were selected. Only patients with triple-negative MBC were eligible for inclusion in the comparative cohort. Thirty-five patients had MBC, and 103 had TN-IBC. All T, N, and overall TNM stage variables used in the present study, including those incorporated into the propensity score matching model, were based on pathological staging.

Histopathological diagnoses were obtained from the original pathology reports of patients diagnosed between 2003 and 2024. Because the study period spanned several editions of the World Health Organization (WHO) classification, diagnoses were originally rendered according to the pathological criteria and institutional standards applicable at the time. For the present study, metaplastic breast carcinoma (MBC) was defined as an invasive breast carcinoma showing unequivocal squamous and/or mesenchymal differentiation, including spindle-cell, chondroid, osseous, or other heterologous elements, in accordance with the current WHO framework. Mixed metaplastic carcinoma was defined as a tumour containing more than one distinct metaplastic component or a combination of metaplastic and conventional invasive carcinoma components. No minimum percentage of the metaplastic component was required for classification because a specific quantitative cut-off is not defined in the WHO classification. Metaplastic carcinoma with mesenchymal differentiation was defined by the presence of malignant epithelial carcinoma associated with heterologous mesenchymal differentiation, including chondroid, osseous, rhabdomyoid, or other sarcomatoid/heterologous elements. Chondroid differentiation referred to the presence of cartilaginous or chondromyxoid matrix-producing areas, whereas osseous differentiation referred to areas showing malignant bone or osteoid formation. In diagnostically challenging spindle-cell lesions, epithelial differentiation was supported, when available in the original diagnostic work-up, by immunohistochemical expression of epithelial or basal/myoepithelial markers such as pancytokeratin, high-molecular-weight cytokeratins/CK5/6, and p63. Cases classified historically as invasive ductal carcinoma were considered invasive breast carcinoma of no special type (IBC-NST) according to current terminology. ER, PR, and HER2 results were abstracted from the original pathology reports and were based on validated immunohistochemical and/or in situ hybridisation methods used at the time of diagnosis. ER and PR negativity corresponded to <1% nuclear staining, while HER2-negative disease was defined according to the applicable ASCO/CAP recommendations as IHC 0/1+ or absence of HER2 amplification by in situ hybridisation in equivocal cases. Histological grade was recorded as reported by the original pathologist and was not used as a defining diagnostic criterion for MBC. Central histopathological re-review was not performed; diagnoses were based on the original pathology reports.

Propensity scores were estimated using logistic regression with histological group as the dependent variable and age and pathological TNM stage as prespecified covariates. Propensity score matching was performed in R version 3.6.3 (R Foundation for Statistical Computing, Vienna, Austria) using the MatchIt 3.0.2 package. Age and TNM stage were selected a priori as matching variables because they represent established prognostic characteristics and were consistently available throughout the long retrospective study period. Histological grade was not included in the propensity score model because grading is not directly comparable across metaplastic breast carcinoma and conventional invasive breast carcinoma of no special type, owing to the marked morphological heterogeneity of MBC. Overall pathological TNM stage, rather than the individual T and N components, was included in the propensity score model as a prespecified summary measure of anatomical disease burden. This approach was chosen to maintain model parsimony in view of the limited number of MBC cases and to avoid introducing closely related staging variables simultaneously into the propensity score model. Because TNM stage integrates information from both primary tumour extent and regional nodal involvement and is a well-established prognostic determinant in early breast cancer, it was considered an appropriate matching variable for the primary comparison. Nevertheless, we acknowledge that MBC may exhibit a distinct pattern characterised by relatively larger primary tumours and less frequent nodal involvement; therefore, matching on overall TNM stage may not fully eliminate imbalance in the individual T and N components. In addition, given the small number of MBC cases, inclusion of additional covariates could have impaired common support and reduced the number of successfully matched pairs. Histological grade, T stage and lymph-node status were therefore retained as clinicopathological variables for post-matching descriptive comparison rather than as a matching covariate.

Patients with metaplastic breast carcinoma were matched 1:1 to patients with triple-negative invasive breast carcinoma using nearest-neighbour propensity score matching without replacement. No caliper restriction was applied. Covariate balance before and after matching was assessed using standardized mean differences (SMDs), with an absolute SMD < 0.10 considered indicative of adequate balance. Of the initial 35 patients with MBC and 103 patients with triple-negative invasive breast carcinoma, all 35 patients with MBC were successfully matched with 35 control patients, resulting in a final matched cohort of 70 patients. After matching, age showed excellent post-matching balance, whereas small residual imbalances remained in stage I and stage II disease (absolute SMDs 0.136 and 0.131, respectively).

The NPI was calculated for each patient as 0.2 × tumour size in centimetres + lymph-node stage + histological grade [10]. Lymph-node stage was coded as 1 for node-negative disease, 2 for one to three positive nodes, and 3 for four or more positive nodes; histological grade was coded from 1 to 3. Higher NPI values indicate a less favourable prognosis. In the present analysis, NPI was treated as a continuous variable and compared between the MBC and TN-IBC groups.

The distribution of continuous variables was assessed using visual methods and the Shapiro–Wilk test. Continuous variables were expressed as median and interquartile range, while categorical variables were presented as number and percentage. Comparisons between patients with TN-IBC and MBC were performed using the Mann–Whitney U test for continuous variables. Categorical variables were compared using the chi-square test or Fisher’s exact test, as appropriate. NLR was evaluated both as a continuous variable and as a dichotomous variable according to the median value of the overall cohort. Disease-free survival (DFS) was defined as the interval from surgery date to recurrence or death, while overall survival (OS) was from breast cancer diagnosis to death. Survival analyses were performed for disease-free survival and overall survival. Because propensity score matching generated a 1:1 matched cohort, the dependence between patients within matched pairs was taken into account in the survival analyses. Cox proportional hazards regression models with robust sandwich variance estimates clustered by matched pair were used to estimate hazard ratios (HRs) and 95% confidence intervals (CIs) for DFS and OS. Triple-negative invasive breast carcinoma was used as the reference group. Kaplan–Meier estimates were used to describe survival probabilities, whereas statistical inference regarding the effect of histological group was based on the pair-clustered robust Cox models. The prognostic effect of NLR was evaluated in the overall cohort and separately in the MBC subgroup. Five-year survival rates were estimated using Kaplan–Meier analysis. All statistical tests were two-sided, and a *p* value of <0.05 was considered statistically significant.

The study was conducted in accordance with the Declaration of Helsinki and approved by the Institutional Review Board of BURSA CITY HOSPITAL (protocol code 2026-05/14 and date of approval: 4 March 2026) for studies involving humans.

## 3. Results

The study included 70 patients (35 MBC, 35 TN-IBC), all female and with triple-negative breast cancer (ER, PR, HER2 negative). MBC subtypes were: squamous cell carcinoma (48.6%, *n* = 17), mesenchymal (14.3%, *n* = 5), spindle cell carcinoma (20.0%, *n* = 7), and mixed histology (17.1%, *n* = 6). After the propensity score matching, a small residual imbalance was observed in individual TNM-stage categories, although the overall pathological stage distributions were highly comparable between groups (Table 1).

The baseline clinicopathological characteristics of the study groups are summarized in Table 2. Age, menopausal status, tumor size, Ki-67 index, T stage, lymph node positivity, pathological stage, and NPI were comparable between the two groups. Grade 3 disease was more frequent in the TN-IBC group than in the MBC group [32 (91.4%) vs. 25 (71.4%), *p* = 0.031]. Axillary dissection was also more frequently performed in the TN-IBC group [19 (54.3%) vs. 8 (22.9%), *p* = 0.007]. No significant differences were observed between the groups in terms of neoadjuvant/adjuvant treatment or radiotherapy. NLR was significantly higher in patients with MBC than in those with TN-IBC [2.31 (1.87–2.65) vs. 1.85 (1.53–2.26), *p* = 0.019]. However, the monocyte-to-lymphocyte ratio, platelet-to-lymphocyte ratio, and pan-immune-inflammation value did not differ significantly between the two groups.

Median follow-up was 7.7 years (range 0.25–20 years). Recurrence occurred in 14 patients (20.0%), recurrence/death in 17 patients (24.3%), and death in 12 patients (17.1%). Recurrences were observed in 8 patients (22.9%) in the MBC group and 6 (17.1%) in the TN-IBC group; deaths occurred in 9 MBC patients (25.7%) and 3 TN-IBC patients (8.6%). Median DFS and OS were not reached. The proportion of recurrence-free patients at 2, 5, and 10 years was 82.8%, 73.9%, and 69.3% in the MBC group and 97.1%, 85.2%, and 76.8% in the TN-IBC group, respectively, with no significant DFS difference (unadjusted log-rank *p* = 0.276). The proportion of patients surviving at 2, 5, and 10 years was 85.7%, 79.8%, and 71.8% in the MBC group and 97.1%, 94.3%, and 90.4% in the TN-IBC group, respectively, with worse OS noted in the MBC group (unadjusted log-rank *p* = 0.044).

The prognostic effect of NLR on survival outcomes is shown in Table 3. In the overall matched cohort, NLR was not significantly associated with DFS when analysed as a continuous variable (HR = 1.01, 95% CI 0.77–1.32; *p* = 0.963) or after dichotomisation at the cohort median, which is 2.080 (HR = 1.65, 95% CI 0.74–3.70; *p* = 0.220). Similarly, continuous NLR was not significantly associated with OS (HR = 1.14, 95% CI 0.87–1.48; *p* = 0.348). However, patients with NLR above the cohort median had a higher mortality hazard than those with lower NLR values in the Cox model using robust variance estimates clustered by matched pair (HR = 3.36, 95% CI 1.07–10.57; *p* = 0.038). In the MBC subgroup, NLR was not significantly associated with either DFS or OS, whether analysed continuously or dichotomised. Given the small number of deaths and the wide confidence interval, the association between high NLR and OS in the overall cohort should be interpreted cautiously and considered exploratory.

Survival outcomes according to histological group are presented in Table 4. In the matched-pair analysis, DFS events occurred in 7 patients (20.0%) in the TN-IBC group and 10 patients (28.6%) in the MBC group. The 5-year DFS rate was 85.2% in the TN-IBC group and 73.9% in the MBC group. MBC was associated with a numerically higher risk of a DFS event; however, the difference was not statistically significant in the Cox model using robust variance estimates clustered by matched pair (HR = 1.70, 95% CI 0.67–4.30; *p* = 0.262). Overall survival events occurred in 3 patients (8.6%) with TN-IBC and 9 patients (25.7%) with MBC. The 5-year OS rate was 94.3% in the TN-IBC group and 79.8% in the MBC group. In the pair-clustered robust Cox model, metaplastic histology was associated with a significantly higher mortality hazard compared with TN-IBC (HR = 3.53, 95% CI 1.01–12.41; *p* = 0.049). Nevertheless, the wide confidence interval reflects the small number of deaths and warrants cautious interpretation of the magnitude of this association.

## 4. Discussion

In this 1:1 propensity score-matched comparison of metaplastic breast carcinoma (MBC) and triple-negative invasive breast carcinoma (TN-IBC), MBC was associated with poorer overall survival (OS) after matching for age and TNM stage. Five-year OS was 79.8% in the MBC group and 94.3% in the TN-IBC group, and the pair-clustered robust Cox model demonstrated a higher mortality hazard for MBC (HR = 3.53, 95% CI 1.01–12.41; *p* = 0.049). By contrast, disease-free survival (DFS) did not differ significantly between groups (HR = 1.70, 95% CI 0.67–4.30; *p* = 0.262). The poorer OS observed in patients with MBC is consistent with previous comparisons of MBC and TN-IBC. Jung et al. and Bae et al. reported inferior survival for MBC despite comparisons with receptor-matched or clinically comparable TN-IBC cohorts [5,9]. Similar findings have been reported in population-based and national database studies [17,18].

DFS was numerically poorer in MBC, with 5-year rates of 73.9% versus 85.2% and event rates of 28.6% versus 20.0% for MBC and TN-IBC, respectively. The absence of statistical significance should not be interpreted as evidence of equivalent recurrence risk, because only 17 DFS events occurred. The divergence between OS and DFS may reflect differences in post-recurrence outcomes or competing causes of death; however, cause-specific mortality and detailed post-recurrence treatment data were unavailable.

Several clinicopathological and treatment differences warrant consideration. Grade 3 disease was more frequent in TN-IBC than in MBC, yet OS was poorer in the MBC group. This observation is compatible with the concept that conventional histological grade may not fully capture the biological aggressiveness of metaplastic differentiation; however, grade was not included in the matching model, and this interpretation remains exploratory. Although previous cohorts have documented low pathological complete response rates with conventional neoadjuvant therapy in MBC [8,9,19], pathological response was not evaluated in the present study; therefore, no conclusion regarding chemotherapy resistance can be drawn from our data.

The significant difference in the frequency of axillary lymph node dissection between the two groups warrants consideration. Axillary dissection was performed more frequently in patients with TN-IBC than in those with MBC, despite comparable rates of lymph node-positive disease. This imbalance may partly reflect differences in surgical practice over the retrospective study period, as well as the recognised tendency of MBC to demonstrate relatively less frequent axillary involvement despite its aggressive clinical behaviour. Nevertheless, differences in the extent of axillary surgery may have influenced the completeness of nodal assessment and regional disease control and therefore represent a potential source of residual confounding. Although adjustment for axillary dissection in a multivariable Cox model could theoretically address this imbalance, only 12 deaths occurred in the matched cohort, substantially limiting the number of covariates that could be reliably incorporated into the model. Inclusion of multiple additional variables under these conditions would increase the risk of model overfitting and unstable effect estimates. Accordingly, we elected not to construct an extensively adjusted multivariable model, and the potential influence of differences in axillary management should be considered when interpreting the survival findings.

The NPI was comparable between the MBC and TN-IBC groups. Because the index is determined by tumour size, nodal burden, and histological grade, this finding is consistent with the comparable tumour size, lymph-node status, and pathological stage distributions after matching, despite grade 3 disease being more frequent in TN-IBC. Notably, OS remained poorer in MBC, suggesting that a conventional composite based on anatomical extent and grade did not account for the observed survival difference. Although NPI has demonstrated prognostic utility in operable breast cancer and TN-IBC [10,11], it was not modelled as an independent survival covariate in our cohort. The present findings therefore should not be interpreted as evidence that NPI lacks prognostic value in MBC; rather, they indicate that comparable NPI distributions do not exclude clinically relevant biological differences between metaplastic and conventional triple-negative carcinoma.

A further finding of the present study was the significantly higher baseline NLR in patients with MBC than in those with TN-IBC (median 2.31 vs. 1.85; *p* = 0.019), whereas MLR, PLR, and PIV were comparable between the groups. This isolated difference may reflect a distinct systemic inflammatory profile in MBC; however, it does not establish a causal relationship with tumour behaviour. In the overall propensity score-matched cohort, NLR was not associated with DFS or OS when analysed as a continuous variable. However, when dichotomised according to the median value of the matched cohort, high NLR was associated with a significantly higher mortality hazard in the pair-clustered robust Cox model (HR = 3.36, 95% CI 1.07–10.57; *p* = 0.038). This observation is consistent with previous studies reporting an association between elevated pretreatment NLR and adverse survival outcomes in breast cancer, particularly in triple-negative disease [13,14,15]. Nevertheless, the prognostic significance of NLR remains controversial, and a recent large study of early breast cancer found no independent relationship between NLR and survival or consistent association with tumour immune characteristics [16]. Importantly, the association observed in our study was not reproduced when NLR was analysed as a continuous variable and was also absent within the MBC subgroup, where neither DFS nor OS was significantly associated with NLR. Thus, systemic inflammatory status may have prognostic relevance across the overall matched triple-negative cohort, but our findings do not support NLR as an MBC-specific prognostic biomarker. Given the limited number of deaths, wide confidence interval, internally derived median cut-off, and potential influence of unmeasured inflammatory conditions, infections, comorbidities, or medications, this association should be regarded as exploratory and requires validation in larger, independent cohorts using prespecified NLR thresholds.

The adverse clinical course of MBC is biologically plausible given its marked histological and molecular heterogeneity. In our cohort, squamous carcinoma was the most frequent subtype, followed by spindle cell, mixed, and mesenchymal variants [20,21]. Molecular studies have identified recurrent alterations involving the PI3K/AKT/mTOR, EGFR, and Wnt pathways, while PD-L1 expression has been reported in a substantial proportion of metaplastic tumours [2,22]. These observations provide a rationale for molecularly selected therapies and immune checkpoint inhibition. Nevertheless, clinical activity reported in broader metastatic TNBC populations cannot be directly extrapolated to MBC [23], and prospective MBC-specific evidence remains limited [24]. Therapeutic implications should therefore be framed as a research priority rather than as a conclusion of the present analysis.

The strengths of this study include the use of propensity score matching to improve balance for age and stage, together with a median follow-up of 7.7 years. Several limitations remain. The retrospective design precludes causal inference, and propensity matching cannot eliminate residual or unmeasured confounding. The cohort was small, with few recurrence and death events, resulting in limited statistical power and imprecise hazard ratios. Treatment extended from 2003 to 2024, during which systemic therapy, surgery, radiotherapy, pathological classification, and supportive care evolved. Numerical imbalances in grade and axillary surgery persisted after matching. Treatment era was not incorporated into the propensity-score or survival models because of the limited cohort size and number of outcome events; therefore, residual confounding related to temporal changes in treatment cannot be excluded. In patients who received neoadjuvant therapy, pathological staging reflected post-treatment ypTNM status; therefore, the use of pathological TNM stage in the propensity-score model may have introduced residual bias because this variable could have been influenced by treatment response rather than representing purely pretreatment disease burden. Data on cause of death, pathological response to neoadjuvant therapy, post-recurrence treatment, PD-L1 status, and genomic alterations were unavailable. Finally, the NLR cut-off was derived from the study cohort and was not externally validated.

## 5. Conclusions

In this propensity score-matched cohort, metaplastic breast carcinoma was associated with poorer overall survival compared with triple-negative invasive breast carcinoma, while disease-free survival did not differ significantly between the groups. Pair-clustered robust Cox analysis confirmed a higher mortality hazard for MBC, although the limited number of deaths and the wide confidence interval warrant cautious interpretation. Baseline NLR was significantly higher in MBC, and a high NLR was associated with poorer overall survival in the overall matched cohort; however, this association was not observed when NLR was analysed as a continuous variable or within the MBC subgroup. These findings suggest that MBC remains a clinically distinct high-risk entity and that systemic inflammatory status may have prognostic relevance across triple-negative breast cancer, but NLR cannot yet be considered an MBC-specific biomarker. Given the retrospective design, small sample size, limited event number, and residual treatment and surgical heterogeneity, the results should be regarded as hypothesis-generating. Larger multicentre studies incorporating standardised inflammatory markers, detailed treatment-response data, and molecular profiling are needed to validate these findings and refine risk stratification in MBC.

## Figures and Tables

**Table 1:** Covariate Balance Before and After Propensity Score Matching Assessed by Standardized Mean Differences.

	SMD Before Matching	SMD After Matching
Age	0.133	0.008
TNM stage 1	0.777	0.136
TNM stage 2	0.679	0.131
TNM stage 3	0.029	0.000

SMD: standardized mean differences. An absolute SMD < 0.10 was considered indicative of adequate covariate balance.

**Table 2:** Baseline clinicopathological characteristics according to histological group.

Variables	TN-IBC, *n* = 35	MBC, *n* = 35	*p* Value
Age, years	50.35 (42.37–58.87)	49.00 (44.50–60.00)	0.796
Postmenopausal status, *n* (%)	23 (65.7)	16 (45.7)	0.092
Tumor size, cm	2.50 (2.00–3.40)	2.50 (1.20–3.30)	0.273
Ki-67, %	45.00 (40.00–60.00)	40.00 (33.75–62.50)	0.508
Histological grade, *n* (%)			**0.031**
Grade 2	3 (8.6)	10 (28.6)	
Grade 3	32 (91.4)	25 (71.4)	
Pathological T stage, *n* (%)			0.780
T1	12 (34.3)	15 (42.9)	
T2	21 (60.0)	17 (48.6)	
T3	1 (2.9)	2 (5.7)	
T4	1 (2.9)	1 (2.9)	
Lymph node-positive disease, *n* (%)	12 (34.3)	11 (31.4)	0.799
Pathological stage, *n* (%)			0.849
Stage I	9 (25.7)	7 (20.0)	
Stage II	25 (71.4)	27 (77.1)	
Stage III	1 (2.9)	1 (2.9)	
Nottingham prognostic index	4.70 (4.40–5.48)	4.54 (4.20–5.24)	0.287
Mastectomy, *n* (%)	2 (5.7)	7 (20.0)	0.151
Axillary dissection, *n* (%)	19 (54.3)	8 (22.9)	**0.007**
Neoadjuvant treatment, *n* (%)	8 (22.9)	12 (34.3)	0.290
Adjuvant treatment, *n* (%)	27 (77.1)	21 (60.0)	0.122
Radiotherapy, *n* (%)	32 (91.4)	31 (88.6)	1.000
NLR	1.85 (1.53–2.26)	2.31 (1.87–2.65)	**0.019**
MLR	0.27 (0.20–0.33)	0.28 (0.22–0.37)	0.411
PLR	127.27 (102.77–155.12)	130.58 (108.47–157.06)	0.760
PIV	230.71 (159.87–464.43)	298.86 (192.51–426.71)	0.565

TN-IBC: Triple-negative invasive breast carcinoma; MBC: metaplastic breast carcinoma; NLR: neutrophil-to-lymphocyte ratio; MLR: monocyte-to-lymphocyte ratio; PLR: platelet-to-lymphocyte ratio; PIV: pan-immune-inflammation value.

**Table 3:** Prognostic impact of NLR on DFS and OS in the overall cohort and metaplastic breast carcinoma subgroup.

Cohort	Endpoint	*n*/Events	NLR as Continuous Variable HR 95% CI	*p* Value	High NLR vs. Low NLR HR 95% CI	*p* Value
Overall cohort	DFS	70/17	1.01 (0.77–1.32)	0.963	1.65 (0.74–3.70)	0.220
Overall cohort	OS	70/12	1.14 (0.87–1.48)	0.348	**3.36 (1.07–10.57)**	**0.038**
Metaplastic group	DFS	35/10	0.93 (0.57–1.51)	0.773	2.60 (0.55–12.29)	0.227
Metaplastic group	OS	35/9	0.93 (0.56–1.55)	0.778	2.14 (0.44–10.33)	0.345

Overall cohort: robust sandwich variance clustered by propensity score-matched pair. High NLR was defined as NLR > 2.08, based on the median of the overall matched cohort. DFS: disease-free survival; OS: overall survival; NLR: neutrophil-to-lymphocyte ratio, CI: confidence interval.

**Table 4:** Survival Outcomes According to Histological Group Using Matched-Pair Cluster-Robust Cox Regression.

Endpoint	TN-IBC, *n* = 35	MBC, *n* = 35	HR 95% CI	Robust *p* Value
DFS events, *n* (%)	7 (20.0)	10 (28.6)	1.70 (0.67–4.30)	0.262
5-year DFS rate, %	85.2	73.9		
OS events, *n* (%)	3 (8.6)	9 (25.7)	3.53 (1.01–12.41)	**0.049**
5-year OS rate, %	94.3	79.8		

HR > 1 indicates a higher hazard for MBC relative to TN-IBC. Robust standard errors were clustered by propensity score-matched pair. DFS: disease-free survival; OS: overall survival; TN-IBC: triple-negative invasive breast carcinoma; MBC: metaplastic breast carcinoma; HR: hazard ratio; CI: confidence interval.

## Data Availability

The research data can be obtained from the corresponding author upon reasonable request.

## Abbreviations

The following abbreviations are used in this manuscript:

MBC	Metaplastic breast carcinoma
IBC-NST	Invasive breast carcinoma of no special type
TN-IBC	Triple-negative invasive breast carcinoma
ER	Estrogen receptor
PR	Progesterone receptor
HER2	Human epidermal growth factor receptor 2
NLR	Neutrophil-to-lymphocyte ratio
MLR	Monocyte-to-lymphocyte ratio
PLR	Platelet-to-lymphocyte ratio
PIV	Pan-immune-inflammation value
NPI	Nottingham Prognostic Index
DFS	Disease-free survival
OS	Overall survival
HR	Hazard ratio
CI	Confidence interval

## References

[1] Hoon Tan P., Ellis I., Allison K., Brogi E., Fox S.B., Lakhani S., Lazar A.J., Morris E.A., Sahin A., Salgado R. (2020). The 2019 World Health Organization classification of tumours of the breast. Histopathology.

[2] Reed A.E.M., Kalaw E., Nones K., Bettington M., Lim M., Bennett J., Johnstone K., Kutasovic J.R., Saunus J.M., Kazakoff S. (2019). Phenotypic and molecular dissection of metaplastic breast cancer and the prognostic implications. J. Pathol..

[3] Pezzi C.M., Patel-Parekh L., Cole K., Franko J., Klimberg V.S., Bland K. (2007). Characteristics and treatment of metaplastic breast cancer: Analysis of 892 cases from the National Cancer Data Base. Ann. Surg. Oncol..

[4] National Cancer Institute Surveillance, Epidemiology, and End Results Program (SEER) (2024). Breast Cancer Subtypes. https://seer.cancer.gov/statfacts/html/breast-subtypes.html.

[5] Jung S.-Y., Kim H.Y., Nam B.-H., Min S.Y., Lee S.J., Park C., Kwon Y., Kim E.-A., Ko K.L., Shin K.H. (2010). Worse prognosis of metaplastic breast cancer patients than other patients with triple-negative breast cancer. Breast Cancer Res. Treat..

[6] Dent R., Trudeau M., Pritchard K.I., Hanna W.M., Kahn H.K., Sawka C.A., Lickley L.A., Rawlinson E., Sun P., Narod S.A. (2007). Triple-negative breast cancer: Clinical features and patterns of recurrence. Clin. Cancer Res. Off. J. Am. Assoc. Cancer Res..

[7] Cimino-Mathews A., Verma S., Figueroa-Magalhaes M.C., Jeter S.C., Zhang Z., Argani P., Stearns V., Connolly R.M. (2016). A Clinicopathologic Analysis of 45 Patients with Metaplastic Breast Carcinoma. Am. J. Clin. Pathol..

[8] Song Y., Liu X., Zhang G., Song H., Ren Y., He X., Wang Y., Zhang J., Zhang Y., Sun S. (2013). Unique clinicopathological features of metaplastic breast carcinoma compared with invasive ductal carcinoma and poor prognostic indicators. World J. Surg. Oncol..

[9] Bae S.Y., Lee S.K., Koo M.Y., Hur S.M., Choi M.-Y., Cho D.H., Kim S., Choe J.-H., Lee J.E., Kim J.-H. (2011). The prognoses of metaplastic breast cancer patients compared to those of triple-negative breast cancer patients. Breast Cancer Res. Treat..

[10] Galea M.H., Blamey R.W., Elston C.E., Ellis I.O. (1992). The Nottingham Prognostic Index in primary breast cancer. Breast Cancer Res. Treat..

[11] Albergaria A., Ricardo S., Milanezi F., Carneiro V., Amendoeira I., Vieira D., Cameselle-Teijeiro J., Schmitt F. (2011). Nottingham Prognostic Index in triple-negative breast cancer: A reliable prognostic tool?. BMC Cancer.

[12] Sejben A., Nyári T., Zombori T., Cserni G. (2020). Comparison of Nottingham Prognostic Index, PREDICT and PrognosTILs in Triple Negative Breast Cancer: A Retrospective Cohort Study. Pathol. Oncol. Res..

[13] Azab B., Bhatt V.R., Phookan J., Murukutla S., Kohn N., Terjanian T., Widmann W.D. (2012). Usefulness of the neutrophil-to-lymphocyte ratio in predicting short- and long-term mortality in breast cancer patients. Ann. Surg. Oncol..

[14] Pistelli M., De Lisa M., Ballatore Z., Caramanti M., Pagliacci A., Battelli N., Ridolfi F., Santoni M., Maccaroni E., Bracci R. (2015). Pre-treatment neutrophil to lymphocyte ratio may be a useful tool in predicting survival in early triple negative breast cancer patients. BMC Cancer.

[15] Jia W., Wu J., Jia H., Yang Y., Zhang X., Chen K., Su F. (2015). The peripheral blood neutrophil-to-lymphocyte ratio is superior to the lymphocyte-to-monocyte ratio for predicting the long-term survival of triple-negative breast cancer patients. PLoS ONE.

[16] Garcia-Torralba E., Ramos M.P., Rubio A.I., Manzano E.N., Boluda N.B., Gil M.L., Aller A., Barrio P.D.L.M., Garre E.G., Díaz F.M. (2024). Deconstructing neutrophil to lymphocyte ratio (NLR) in early breast cancer: Lack of prognostic utility and biological correlates across tumour subtypes. Breast Cancer Res. Treat..

[17] Nelson R.A., Guye M.L., Luu T., Lai L.L. (2015). Survival outcomes of metaplastic breast cancer patients: Results from a US population-based analysis. Ann. Surg. Oncol..

[18] Ong C.T., Campbell B.M., Thomas S.M., Greenup R.A., Plichta J.K., Rosenberger L.H., Force J., Hall A., Hyslop T., Hwang E.S. (2018). Metaplastic Breast Cancer Treatment and Outcomes in 2500 Patients: A Retrospective Analysis of a National Oncology Database. Ann. Surg. Oncol..

[19] Al-Hilli Z., Choong G., Keeney M.G., Visscher D.W., Ingle J.N., Goetz M.P., Jakub J.W. (2019). Metaplastic breast cancer has a poor response to neoadjuvant systemic therapy. Breast Cancer Res. Treat..

[20] Rakha E.A., Tan P.H., Varga Z., Tse G.M., Shaaban A.M., Climent F., van Deurzen C.H.M., Purnell D., Dodwell D., Chan T. (2015). Prognostic factors in metaplastic carcinoma of the breast: A multi-institutional study. Br. J. Cancer.

[21] Zhang Y., Lv F., Yang Y., Qian X., Lang R., Fan Y., Liu F., Li Y., Li S., Shen B. (2015). Clinicopathological Features and Prognosis of Metaplastic Breast Carcinoma: Experience of a Major Chinese Cancer Center. PLoS ONE.

[22] Joneja U., Vranic S., Swensen J., Feldman R., Chen W., Kimbrough J., Xiao N., Reddy S., Palazzo J., Gatalica Z. (2017). Comprehensive profiling of metaplastic breast carcinomas reveals frequent overexpression of programmed death-ligand 1. J. Clin. Pathol..

[23] Adams S., Diamond J.R., Hamilton E., Pohlmann P.R., Tolaney S.M., Chang C.-W., Zhang W., Iizuka K., Foster P.G., Molinero L. (2019). Atezolizumab Plus nab-Paclitaxel in the Treatment of Metastatic Triple-Negative Breast Cancer with 2-Year Survival Follow-up: A Phase 1b Clinical Trial. JAMA Oncol..

[24] Tray N., Taff J., Adams S. (2019). Therapeutic landscape of metaplastic breast cancer. Cancer Treat. Rev..

